# Associations of Perfluoroalkyl substances (PFAS) with terminal ductal lobular unit involution of the normal breast

**DOI:** 10.21203/rs.3.rs-6829962/v1

**Published:** 2025-07-02

**Authors:** Katherine W. Reeves, Youssef Oulhote, Philippe Grandjean, Flemming Nielsen

**Affiliations:** University of Massachusetts Amherst; Mt. Sinai Icahn School of Medicine, Icahn School of Medicine at Mount Sinai; National Institute of Public Health, University of Southern Denmark; University of Southern Denmark

**Keywords:** Per- and polyfluoroalkyl substances (PFAS), involution, terminal ductal lobular unit

## Abstract

**Background:**

Per- and polyfluoroalkyl substances (PFAS) may be carcinogenic, and animal studies demonstrate their harmful effects on mammary gland development. Terminal ductal lobular units (TDLUs) are the structures that produce milk following childbirth, and involution of TDLUs normally occurs with aging. Most breast cancers arise from TDLUs, and a greater degree of TDLU involution is associated with lower breast cancer risk. We estimated associations between PFAS concentrations and TDLU involution in normal breast tissue samples.

**Methods:**

Concentrations of seven PFAS were measured in serum provided by a subset of 263 healthy volunteer participants from the Susan G. Komen for the Cure Tissue Bank (KTB) who were postmenopausal, not currently using hormone therapy, and had available TDLU measurements. Bayesian kernel machine regression and quantile-G computation were used to estimate covariate-adjusted associations between the PFAS mixture and measures of TDLU involution (presence of TDLUs, number of observed TDLUs, and median TDLU span) within this population and with stratification on parity and breastfeeding history.

**Results:**

Distributions of PFAS were similar between participants with (N = 106) and without (N = 157) observed TDLUs. No strong, statistically significant associations were observed between individual PFAS and presence of observed TDLUs. The overall effect of the PFAS mixture suggested an inverted U-shaped association with odds of observed TDLUs, although this was not statistically significant. Among the subgroup of parous women, stratified analyses suggested a positive association between the PFAS mixture and observed TDLUs among those who had ever breastfed, but a slightly negative association among those who had never breastfed.

**Conclusions:**

Overall, our analysis does not support meaningful effects of PFAS on TDLU involution, although we note that these findings are not applicable to premenopausal women or to postmenopausal women using hormone therapy.

## Background

Per- and polyfluoroalkyl substances (PFAS) have detrimental effects on the mammary gland in experimental studies (1–5). International Agency for Research on Cancer identifies the two most common PFAS, perfluorooctanoic acid (PFOA) and perfluorooctane sulfate (PFOS), as carcinogenic and possibly carcinogenic, respectively (6). PFAS are widely used in non-stick, water-resistant, and stain-resistant coatings for consumer products. Biomonitoring studies report detectable levels of PFAS in > 98% of the U.S. population (7). PFAS accumulate in human tissues and may have half-lives of many years (8–10), prompting major concerns about their potential for long-term health effects. Although not genotoxic (11), they may interact with peroxisome proliferator-activated receptor-α and also interact with steroid hormones (11); these mechanisms are particularly concerning as each may stimulate carcinogenic processes.

Accumulating evidence suggests that PFAS exposure could promote breast cancer. Experiments using mouse models show that perfluorooctanoic acid (PFOA) exposure delays mammary gland development (2). Separate studies showed that these effects can persist across three generations (3, 5) and may occur even at low-dose exposures (4). PFOA and PFOS are both found in human breast milk (8, 12, 13). Consistent inverse associations between circulating PFAS concentrations and breastfeeding duration(14, 15) may indicate deleterious effects of PFAS on normal breast development and function, in agreement with experimental findings. Prior epidemiologic studies offer preliminary evidence that exposure to certain PFAS may be associated with a doubling of breast cancer risk (16–18), although null associations have been reported by other studies (17, 19, 20). Thus, whether PFAS exposure contributesto human breast cancer risk remains unclear.

Exploring features of human breast tissue can provide important insights on breast carcinogenesis and the impacts of specific exposures. Terminal ductal lobular units (TDLUs) are the structures that produce milk following childbirth, and involution of TDLUs normally occurs with aging. Most breast cancers arise from TDLUs, and a greater degree of TDLU involution (e.g., fewer TDLUs) is associated with lower breast cancer risk (21).

To explore potential associations between PFAS concentrations and breast carcinogenesis, we leveraged prior measurements of TDLU involution from breast tissue samples provided by healthy volunteer participants from the Susan G. Komen for the Cure Tissue Bank (KTB).

## Methods

### Study Population

The Susan G. Komen for the Cure Tissue Bank (KTB) at the Indiana University Simon Cancer Center was established in 2007 to document the molecular histology of the normal breast.(22) Volunteer participants provided epidemiologic data and blood specimens and underwent a breast biopsy to obtain breast tissue samples. To be eligible to donate tissue to the KTB, donors met the following criteria: 1) biological female, 2) age ≥ 18 year, 3) no breast implants, 4) no known allergy to local anesthetics, 5) not currently taking a blood thinner except aspirin, and 6) no prior chest radiation. Data on KTB participants are publicly available to researchers through the Virtual Tissue Bank (https://virtualtissuebank.iu.edu).

We identified KTB participants who met the following eligibility criteria: 1) no history of breast cancer or ductal carcinoma in situ, 2) postmenopausal, 3) no current hormone therapy (HT) use, 4) available serum sample, 5) available postmenopausal mammogram(s), and 6) available postmenopausal TDLU involution measurements. We further excluded participants who were subsequently diagnosed with breast cancer (n = 14), who were identified to have a rare genetic mutation related to breast cancer risk (n = 4), or who had an unusable quality serum specimen (n = 1), as these samples were not provided by the KTB. The present analysis included 263 participants.

All KTB participants provided written informed consent at the time of donation. Human subjects approval for the KTB was provided by the Institutional Review Board at Indiana University. The present research utilized fully deidentified data and previously collected biospecimens and thus was determined to not qualify as human subjects research by the Institutional Review Board at the University of Massachusetts Amherst.

### PFAS Measurement

Briefly, at the time of breast biopsy serum samples were obtained from blood drawn into 9mL collection tubes and immediately centrifuged for 15 min at 2000 rcf. The serum layer was then transferred into separate 750 μL aliquots in cryogenic vials and stored at −80°C. 750 uL serum samples were retrieved for selected participants and shipped to Clinical Pharmacology, Pharmacy and Environmental Medicine, University of Southern Denmark for PFAS analysis packed in dry ice via overnight courier. All laboratory staff were masked to participants’ characteristics.

The laboratory measured a panel of seven PFAS using online solid-phase extraction followed by high-pressure liquid chromatography with tandem mass spectrometry (23, 24): perfluorooctanoate (PFOA), perfluorooctanoic sulfonate (PFOS), perfluorohexanoic sulfonate (PFHxS), perfluoroheptane sulfonate (PFHpS), perfluorononaoate (PFNA), perfluorodecanoate (PFDA), and perfluoroundecanoate (PFUnDA). The limit of detection (LOD) was 0.03 ng/ml for all PFAS; values < LOD were set to 0.015 ng/mL for analysis (0% PFOA, 1% PFHxS, 0% PFNA, 5% PFHpS, 0% PFDA, 0% PFOS, 6% PFUnDA). Coefficients of variation (CV) were calculated for each PFAS: PFOA (4.1%), PFOS (9.1%), PFNA (4.7%), PFDA (6.3%), PFHxS (10.1%), PFHpS (13.7%), PFUnDA (5.5%). Samples were randomly distributed through batches. No significant variation across batches was observed (for each PFAS, p > 0.9 ANOVA testing batch effect in quality control samples). The accuracy of the analytical method for PFAS was ensured by participation in the German External Quality Assessment Scheme (G-EQUAS), organized by Social and Environmental Medicine of the University of Erlangen-Nuremberg.

### TDLU Measurements

KTB volunteers provided up to six core samples from the upper outer quadrant of a single breast. Needle biopsies were performed under local anesthetic by a surgeon using a 10-gauge needle. Breast tissue samples were immediately processed following biopsy. One core sample was fixed in 10% buffered formalin and processed as paraffin-embedded blocks, from which a 5 μm section was obtained and stained with hematoxylin and eosin (H&E). Digital images of H&E stained tissues are available in the Virtual Tissue Bank.

Prior work assessed three measures of TDLU involution using the digitized H&E sections: TDLU counts per standardized biopsy, median acini counts per TDLU, and median TDLU span (25, 26). Briefly, a trained pathologist assessed up to ten normal TDLUs to measure: a) the TDLU span, using an electronic ruler to measure in microns, and b) the number of acini per TDLU. The median values for each of these measures across the TDLUs assessed were used in analyses. A section of breast tissue (median 34.5 mm^2^) was inspected and the number of TDLUs observed within that section was recorded as the TDLU count.

We utilized three parametrizations of TDLU involution for analysis as outcomes of interest. First, we created a dichotomous variable indicating any observed TDLUs (no, yes). Second, a variable indicating the number of observed TDLUs was created, with values of 0 for participants without observed TDLUs (integers). Third, among participants with observed TDLUs, we calculated the median TDLU span as the median diameter among the measured TDLUs (continuous). Each of these quantitative measurements is negatively associated with TDLU involution (i.e., *higher* TDLU count, TDLU span, and median number of acini per TDLU demonstrate *less* involution). These measurements were assessed for both intra- and inter-observer agreement and were demonstrated to be highly reliable and highly correlated with qualitative assessments of TDLU involution (25).

### Covariate Data

Additional data were obtained from questionnaires completed by KTB participants at the time of sample donation. Participants self-reported their age, education, race, history of tobacco use, history of pregnancies and live births, breastfeeding history, ages at menarche and menopause, and history of postmenopausal hormone therapy (HT) use. Additionally, body mass index (BMI) was calculated from height and weight measured by a research nurse at the enrollment visit. Lifetime risk Gail score (27) was calculated using self-reported data collected at the time of enrollment.

## Statistical Analysis

We calculated age-adjusted descriptive statistics for all covariates, stratified by any observed TDLUs (no, yes). We calculated geometric means for the measured PFAS and tested for differences between those with and without any observed TDLUs using t tests. SAS (version 9.4) was used for these analyses.

Based on prior knowledge and causal diagrams, we included the following variables as covariates in our analysis: age (continuous), race (White, non-White), lifetime risk Gail score (continuous), BMI (continuous), ever used tobacco (no, yes), age at menarche (continuous), ever pregnant (no, yes), time since last menstrual period (continuous), and ever used postmenopausal HT (no, yes).

We used Bayesian Kernel Machine Regression (BKMR) to statistically explore the individual and joint effects of the chemical mixture of PFAS, using the R package “bkmr”. BKMR is a semi-parametric, supervised approach for the detection of the toxic agent(s) that uses a Bayesian approach to variable selection within a kernel machine regression (28). BKMR allows the visualization of the exposure-response association for each mixture component, while accounting for correlation between mixture components, and it also estimates the multivariable exposure-response function in a flexible way that allows for nonlinear and non-additive effects. Prior to fitting BKMR models, each PFAS measure was first natural log-transformed and then standardized by subtracting its mean and dividing by its standard deviation. We used Quantile-G computation to estimate the joint mixture effect, using the R package “qgcomp.” Adjusting for the aforementioned covariates, the individual positive and negative effects of each PFAS and its contribution to the overall mixture effect were calculated.

We repeated the above analyses (1) restricted to the subgroup of women who had never used postmenopausal HT, and (2) among parous women with stratification on breastfeeding history.

We performed BKMR and Quantile-G computation analyses on the following outcomes: observed TDLUs (none, any), number of TDLUs observed (integers), number of TDLUs observed among those with > 0 observed TDLUs (integers), and median TDLU span among those with any observed TDLUs (continuous). BKMR and Quantile g-computation analyses were performed using R 4.4.2. Additionally, because BKMR does not allow for incorporation of zero-inflated regression, we ran exploratory analyses using negative binomial regression with TDLU count as the outcome and individual PFAS as the exposures using SAS version 9.4 (SAS Institute, Cary, NC).

## Results

The average age of participants was 58 years among both those participants with and without observed TDLUs (Table 1). Participants with observed TDLUs (i.e., less involution of breast tissue) were less frequently of White race (63.1% *vs* 75.5%), more frequently reported a history of pregnancy (87.9% *vs* 77.4%), and had a slightly longer time since last menstrual period (13.5 *vs* 12.3 years).

**Figure 1:**
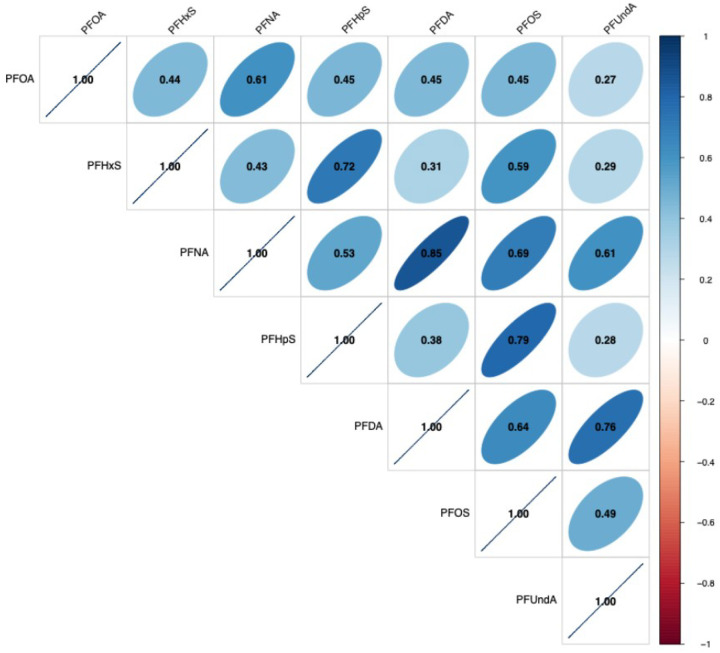
Pairwise correlations between measured PFAS (N=263)

Overall, distributions of PFAS were similar between participants with and without observed TDLUs (Table 2). We observed positive correlations between all measured PFAS (Figure 1). In particular, several strong correlations (r > 0.6) were observed: PFNA and PFOA (r = 0.61), PFHpS and PFHxS (r = 0.72), PFDA and PFNA (r = 0.85), PFOS and PFNA (r = 0.70), PFUndA and PFNA (r = 0.61), PFOS and PFHpS (r = 0.79), PFOS and PFDA (r = 0.64), PFUndA and PFDA (r = 0.76).

BKMR analysis was used to explore the independent and combined effects of the PFAS mixture components (PFOA, PFDA, PFUnDA, PFHpS, PFHxS, PFNA, PFOS) on the outcome of any observed TDLUs (yes *vs* no) (Supplemental Figure 1). Adjusting for other mixture components and covariates, higher concentrations of PFOA increased the odds of any observed TDLUs until approximately the median, after which higher concentrations of PFOA were associated with lower odds of observed TDLUs. PFHxS, PFNA, and PFHpS demonstrated positive associations between concentrations of these chemicals and odds of observed TDLUs. PFUnDA demonstrated a negative association with odds of observed TDLUs. No qualitative associations were observed between PFDA or PFOS and odds of observed TDLUs.

Overall, similar trends were observed when evaluating the effect of each component per one SD increase when setting the other mixture components at specific quantiles, although none were identified as statistically significant (Supplemental Figure 2). In particular, negative associations were observed for PFUnDA and odds of observed TDLUs at the 25^th^, 50^th^, and 75^th^ quantiles of the mixture. Suggested positive associations with odds of observed TDLUs were apparent for PFDA at the 75^th^ quantile of the mixture and for PFHpS and PFHxS at the 25^th^ quantile of the mixture. A negative association between PFOA and odds of observed TDLUs was suggested at the 25^th^ quantile of the mixture. Together, the overall effect of the mixture, adjusting for covariates, suggested an inverted U-shape association between the PFAS mixture and odds of observed TDLUs, whereby both lower mixture concentrations and higher mixture concentrations were negatively associated with odds of observed TDLUs, although these associations did not achieve statistical significance (Supplemental Figure 3). When exploring bivariate interactions, we observed apparent interactions between concentrations of PFUnDA and other PFAS. These plots suggested that the effect of each other mixture component on odds of observed TDLUs was impacted by the concentration of PFUnDA (Supplemental Figure 4).

Next, we repeated analyses with the number of observed TDLUs as the outcome (Supplemental Figures 5–8). Overall, there were no apparent associations between individual PFAS mixture components and number of TDLUs. For the overall mixture effect there was some suggestion of a negative association with number of observed TDLUs, indicating greater involution, although this was not statistically significant. These results were confirmed by the exploratory analyses using negative binomial regression (data not shown).

Because nearly half of the participants had no observed TDLUs (n = 106), we performed further analyses restricted to those participants with observed TDLUs (n = 157). No associations were observed between individual mixture components or the overall mixture and the number of observed TDLUs (data not shown). Among this subgroup, there was some suggestion that PFOS concentrations were negatively associated with the number of TDLUs when the other mixture components were at the 25^th^ percentile, indicating greater involution. There were no apparent associations between the individual mixture components or the overall mixture and median diameter of acini in the observed TDLUs (data not shown).

The above analyses were repeated among the subgroup of participants who did not use postmenopausal HT; results were similar to those observed within the full study population for all outcomes (data not shown).

**Figure 2:**
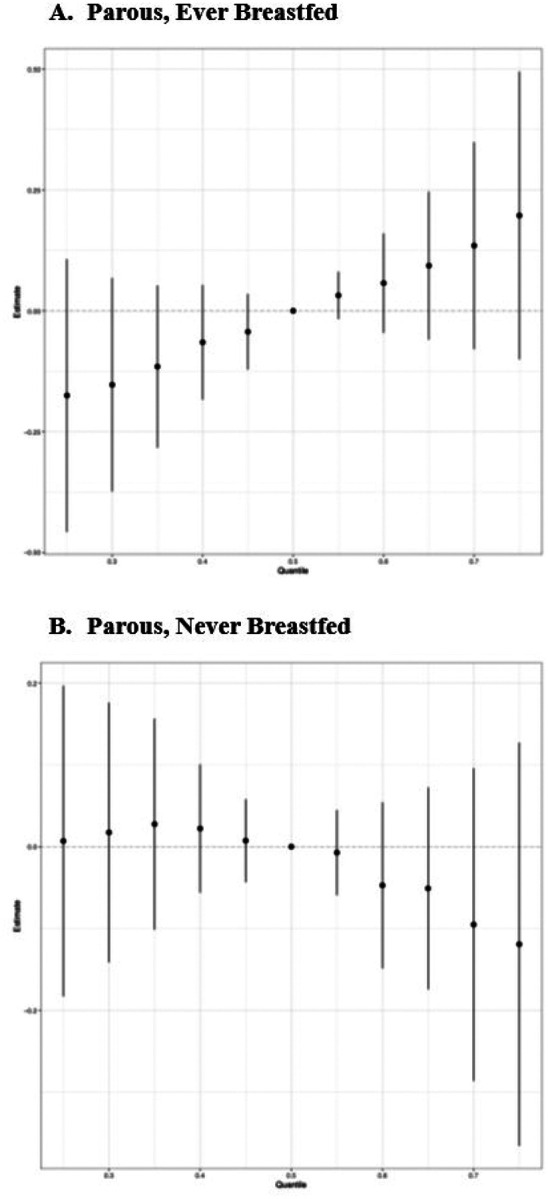
Plots of the overall mixture effect on the outcome of any vs none observed TDLUs, separately (A) among parous women who ever breastfed (N=138) and (B) among parous women who had never breastfed (N=78).

**Figure 3:**
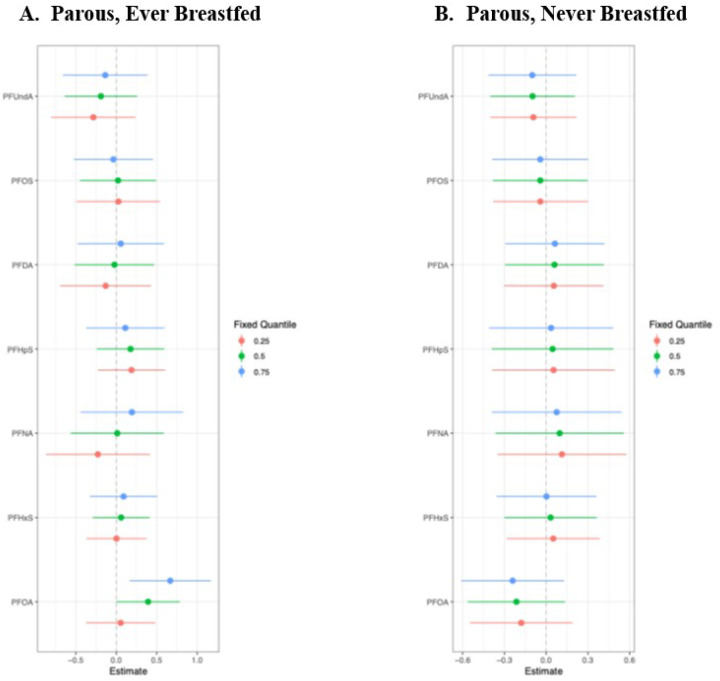
Effects of each PFAS chemical when other mixture components are held at the 25^th^, 50^th^, or 75^th^ percentiles, separately among parous women who (A) ever (N=178) or (B) never (N=78) breastfed.

Next, we explored associations between PFAS and TDLU involution among parous women, with stratification on breastfeeding history. Interestingly, the BKMR analysis identified a positive association between the overall mixture effect and observed TDLUs among parous women who had ever breastfed, indicating less involution (Figure 2A), but a slightly inverse association among parous women who had never breastfed, indicating greater involution (Figure 2B). The difference appeared to be driven by differential associations with PFOA when other mixture components were at the 75^th^ percentile; in these analyses, there was a positive association between PFOA and any observed TDLUs (yes *vs* no) among parous women who had ever breastfeed (Figure 3A), yet a negative association between PFOA and observed TDLUs among parous women who had never breastfed (Figure 3B).

Among the full study population, the results of the Quantile-G computation analysis indicated no overall effect of the PFAS mixture for any of the outcomes we evaluated (Table 3). PFHpS had a consistently positive direction of effect across outcomes, while both PFUnDA and PFDA had consistently negative effects. The direction of effect for the remaining PFAS evaluated (PFOA, PFHxS, PFNA) varied across the four analyses. Similar results were observed among the subgroups of participants who did not use postmenopausal HT and when stratified by breastfeeding history among parous women (data not shown).

## Discussion

Overall, we observed no meaningful associations between serum PFAS concentrations and TDLU involution in this sample of postmenopausal women. Among parous women, stratified analyses using BKMR indicated that the mixture effect may differ by breastfeeding history. These analyses suggested a positive association between PFAS and the presence of TDLUs among those with a history of breastfeeding, yet a negative association among those who had never breastfed. However, the quantile G-computation analysis did not support differential effects by breastfeeding history. The number of nulliparous women were too small to support separate analyses within this subgroup.

This is, to our knowledge, the first study to evaluate potential associations between PFAS and TDLU involution. Recently, higher PM2.5 levels were associated with higher TDLU counts, indicating that greater exposure to this common air pollutant parameter was associated with lower breast tissue involution (29). Such results signal that environmental exposures may have important impacts on breast tissue remodeling and, ultimately, breast cancer risk.

Several epidemiologic studies have explored associations between PFAS and breast cancer risk. A recent meta-analysis of prospective studies (30) estimated a slight, yet non-significant, increase in risk associated with PFOA (4 studies; RR = 1.16, 95% CI 0.96–1.40), but no association with PFOS (4 studies; RR = 1.03, 95% CI 0.87–1.22), PFHxS (2 studies; RR = 0.79, 95% CI 0.51–1.23), or PFNA (2 studies; RR = 1.17, 95% CI 0.86–1.59). However, the relative lack of prospective studies precludes any definitive conclusions. Future research using prospective data and measuring a broad panel of PFAS is needed.

Animal studies provide substantial evidence that PFAS affect breast tissue morphology. PFOA exposure delays mammary gland development in mouse models (2). Separate studies showed that these effects persist across three generations (3, 5) and also result from low-dose exposures (4). Additionally, pregnant mice exposed to PFOA displayed stunted branching of the mammary epithelium and delayed involution (1). Consistent with these deleterious effects on mammary gland development and function, epidemiological studies have reported significant inverse associations between circulating PFAS concentrations and breastfeeding duration (14, 15). Our finding of differential associations among parous women by breastfeeding history may be consistent with this observation. TDLUs produce milk during lactation. If PFAS exposure results in fewer TDLUs, and thus less breastfeeding, then this may appear as greater involution (i.e., lower or no TDLU counts) among those who never breastfed, although their TDLU count initially would have been lower compared to breastfeeding women. Change in TDLU count could add important insight, although we note that prospective repeated measures of TDLU involution are not available and would be extremely challenging to collect in a healthy volunteer population.

The majority of breast cancers arise from TDLUs. Breast tissue changes throughout the life course, and low TDLU involution may foreshadow a future breast cancer diagnosis. Greater TDLU involution in women with benign breast disease (BBD) has been associated with lower breast cancer risk in several studies (21, 31–33), though not all (34). For example, a case-control study of women with BBD observed a non-significant two-fold higher risk among women with the highest TDLU counts compared to those with the lowest (4th *vs* 1st quartile, OR = 2.44, 95% CI 0.96–6.19) (33). However, prior work has not evaluated associations between TDLU involution measures and future breast cancer risk using breast tissue samples from healthy volunteers, similar to the KTB participants studied in the present research. Therefore, the lack of association we observed between PFAS and TDLU involution does not definitively rule out an effect of PFAS on future breast cancer risk. Additionally, TDLUs naturally involute with chronological aging. The lack of association we observed may be due to the older, postmenopausal age of our study population. Future research should assess whether PFAS are associated with TDLU involution in premenopausal women.

Also, while *greater* TDLU involution is associated with *lower* breast cancer risk in women with BBD, prior analyses within the KTB indicate that some factors known to increase breast cancer risk are also associated with greater TDLU involution (25). For example, nulliparous women had lower TDLU counts than women with one live birth (RR = 0.67, 95% CI 0.56–0.97), and increased parity also was positively associated with TDLU counts (RR = 1.20, 95% CI 1.04–1.37), despite parity having a well-established negative association with future breast cancer risk. A similar paradoxical association was observed with breastfeeding history, with the risk of having observed TDLUs estimated to be 24% higher among postmenopausal women with a history of breastfeeding compared to postmenopausal women who had never breastfed (RR = 1.24, 95% CI 1.11–1.38) (25). This finding is consistent with the idea that breastfeeding is associated with more TDLUs, and thus TDLU counts in postmenopausal women may distinguish extent of involution across women only when first stratified by breastfeeding history.

Our findings must be considered in the context of several limitations. KTB donors are majority White and residents of the U.S. state of Indiana. While 32% of our study population was non-White, future research in more racially and ethnically diverse populations is needed. Also, our sample population was limited to postmenopausal women not currently using HT; it is possible that different results would be observed among premenopausal women and among postmenopausal women currently taking HT medications. We acknowledge that we measured PFAS exposure at a single point in time that was coincident with the breast biopsy. However, serum PFAS measurements are accepted as reflecting long-term (~ 5–10 years prior) exposure, thus supporting that measured concentrations of PFAS reflect exposures that preceded the measurements of TDLU involution. Further, while we were specifically powered to test our hypotheses among the full sample population, results of stratified analyses should be interpreted cautiously given the smaller sample sizes and resulting limited statistical power. Finally, the BKMR analysis that was our primary analytic approach did not support modeling TDLU counts, thus we were limited to analyzing these data as any TDLUs observed (yes *vs* no). We ran exploratory analyses using negative binomial regression with TDLU count as the outcome and individual PFAS as the exposures, which confirmed the null associations resulting from the BKMR analyses.

Our analysis was strengthened by many unique methodological features. First, we utilized breast tissue samples donated from a volunteer population of healthy women. Most explorations involving histologic breast tissue measures utilize tissue from breast cancer cases, women undergoing prophylactic mastectomy, or women undergoing reduction mammoplasty. Thus, our findings have potentially greater generalizability to healthy women. Additionally, we measured a broad panel of PFAS, including those with the highest prevalence of exposure in the U.S. population. Because each PFAS may have different effects, it is important to consider compounds beyond PFOA and PFOS, which have received most of the attention in epidemiologic studies. Finally, we utilized statistical analysis approaches that facilitate modeling concentrations of PFAS as a chemical mixture, more closely reflecting human exposures. These analyses allowed us to evaluate both individual effects of each PFAS in the context of exposure to the other PFAS in addition to the effect of the PFAS mixture itself.

## Conclusions

Overall, our analysis does not support meaningful effects of PFAS on TDLU involution, although we note that these findings are not applicable to premenopausal women or to postmenopausal women using HT. Also, it is possible that PFAS promote breast carcinogenesis via a mechanism that does not include effects on TDLU involution. Therefore, the lack of observed association between PFAS and TDLU involution in our analyses does not fully rule out an impact of PFAS on breast cancer risk.

## Supplementary Material

This is a list of supplementary files associated with this preprint. Click to download.

- ReevesPFASTDLUSupplementaryFigures.docx

## Figures and Tables

**Table 1.** Descriptive characteristics of study population by TDLU status (N=263).

	Any TDLUs observed	
	No (N=106)	Yes (N=157)
Age, years; Mean (SD)	58.6 (8.1)	58.7 (7.6)
Education; N (%)		
High School or Less	16 (15.1)	43 (27.4)
Associate’s/Bachelor’s Degree	37 (34.9)	52 (33.1)
Graduate/Professional Degree	39 (36.8)	47 (29.9)
Other/Unknown	14 (13.2)	15 (9.6)
White race; N (%)	80 (75.5)	99 (63.1)
Gail Score (lifetime risk); Mean (SD)	10.3 (5.0)	10.0 (5.5)
Body mass index, kg/m^2^; Mean (SD)	30.6 (7.1)	29.3 (6.7)
Ever used tobacco; N (%)	33 (31.1)	47 (30.0)
Age at menarche, years; Mean (SD)	12.7 (1.4)	12.8 (1.5)
Ever pregnant; N (%)	82 (77.4)	138 (87.9)
Number live births;^a^ N (%)	2.2 (1.2)	2.2 (1.2)
Ever breastfed^a^; N (%)	51 (63.8)	87 (64.0)
Time since last menstrual period, yrs; Mean (SD)	12.3 (10.0)	13.5 (10.4)
Ever used postmenopausal HT; N (%)	38 (35.9)	49 (31.2)
Means (SD) or percentages are standardized to the age distribution of the study population.		

a Among those ever pregnant

**Table 2.** Distribution of serum perfluoroalkyl substance concentrations, by TDLU status (N=263)

	TDLUs Observed				
	No (N=106)		Yes (N=157)		
PFAS Chemical	Geometric Mean (SD)	Min - Max	Geometric Mean (SD)	Min - Max	P value
PFOA, ng/mL	2.64 (2.20)	0.17 – 20.84	2.52 (1.72)	0.41 – 9.95	0.57
PFHxS, ng/mL	0.90 (2.76)	0.02 – 7.19	1.12 (2.30)	0.02 – 7.74	0.06
PFNA, ng/mL	0.76 (1.62)	0.16 – 2.62	0.83 (1.69)	0.10 – 4.47	0.22
PFHpS, ng/mL	0.15 (2.81)	0.02 – 0.97	0.18 (2.43)	0.02 – 1.32	0.12
PFOS, ng/mL	6.99 (2.15)	0.86 – 102.83	7.68 (2.04)	0.23 – 42.36	0.31
PFUnDA, ng/mL	0.10 (2.29)	0.02 – 0.53	0.10 (2.35)	0.02 – 1.07	0.95
PFDA, ng/mL	0.22 (1.67)	0.07 – 0.98	0.23 (1.88)	0.02 – 1.57	0.37

**Table 3.** Summary of estimates of overall mixture effect and individual contribution to the overall mixture effect for each outcome using Quantile-G computation (N=263)^a^

	Any vs None TDLU	Number TDLU	Number TDLU^b^	Median TDLU Span^b^
*Overall Mixture Effect Beta (95% CI); p*				
	0.03 (−2.75, 2.81); p=0.98	−0.22 (−1.20, 0.75); p=0.65	−0.72 (−2.10, 0.65); p=0.31	−4.59 (−22.97, 13.79); p=0.63
	***Individual Weights and Direction of Effect***			
PFOA	0.004	0.21	0.30	−0.29
PFHxS	0.34	0.05	−0.03	−0.36
PFNA	0.18	0.20	−0.34	0.33
PFHpS	0.27	0.54	0.34	0.47
PFOS	0.21	−0.63	−0.46	0.20
PFUnDA	−0.86	−0.26	−0.16	−0.08
PFDA	−0.14	−0.11	−0.35	−0.27

a Adjusted for age, race, body mass index, lifetime Gail risk score, age at menarche, ever been pregnant, years since menopause, ever use of postmenopausal HT

b Among those with observed TDLUs

## Data Availability

Data on Komen Tissue Bank participants are publicly available to researchers through the Virtual Tissue Bank (https://virtualtissuebank.iu.edu).

## Abbreviations

BKMR: Bayesian kernel machine regression
BMI: Body mass index
HT: Hormone therapy
KTB: Komen Tissue Bank
LOD: Limit of detection
PFAS: Per- and polyfluoroalkyl substance
PFDA: Perfluorodecanoate
PFHpS: Perfluoroheptane sulfonate
PFHxS: Perfluorohexanoic sulfonate
PFNA: Perfluorononaoate
PFOA: Perfluoroocatanoic acid
PFOS: Perfluorooctane sulfate
PFUnDA: Perfluoroundecanoate
TDLU: Terminal ductal lobular unit
